# La maladie de Hirayama: à propos de quatre observations tunisiennes et revue de la literature

**DOI:** 10.11604/pamj.2015.20.380.6042

**Published:** 2015-04-16

**Authors:** Sana Ben Amor, Anis Hassine, Ines Chatti, Anissa Khefifi, Mohamed Doggui, Mohamed Salah Harzallah, Sofien Benammou

**Affiliations:** 1 Service de Neurologie, Hôpital Sahloul, Sousse, Tunisie; 2 Service de Neurophysiologie, Hôpital Sahloul, Sousse, Tunisie

**Keywords:** Amyotrophie, corne antérieure, hirayama, Muscular atrophy, anterior horn, hirayama

## Abstract

Nous rapportons les aspects cliniques et électriques de la maladie de Hirayama en Tunisie à travers une série de quatre observations diagnostiquées au service de neurologie Sahloul. Il s'agit de quatre femmes. L’âge moyen était 30,25 ans avec des extrêmes de 27 et 37 ans. Une patiente avait un antécédent de traumatisme cervical, trois avaient une profession favorisant la position prolongée du rachis cervical en flexion. Un déficit moteur distal et une amyotrophie de la main et de l'avant bras droits d'installation progressive étaient observés dans tous les cas. Il n'avait ni des troubles sensitifs objectifs ni de modification des reflexes ostéotendineux et cutanés. L'EMG montrait une dénervation motrice dans le territoire des muscles dépendants des racines C7, C8, et D1. L'IRM cervicale était sans anomalie dans tous les cas. L’évolution était marquée par la bilatéralisation de la symptomatologie chez une patiente et une stabilisation clinique chez les autres. Ainsi, les aspects cliniques et électriques de la maladie de Hirayama dans cette série tunisienne sont comparables à ceux rapportés dans la littérature en dehors d'une atteinte strictement féminine.

## Introduction

La MH a été décrite initialement en 1959 au Japon [1]. C'est une affection rare qui se traduit cliniquement par une atteinte motrice pure réalisant une amyotrophie du membre supérieur unilatéral dans le territoire des myotomes C7 à D1 [2]. Depuis sa première description, quelques séries européennes et américaines ont été aussi publiées [2, 3]. Nous nous proposons de relever les aspects cliniques et paracliniques de cette affection à travers une série de quatre patientes tunisiennes et de les comparer aux données de la littérature.

## Méthodes

Notre travail a porté sur l’étude rétrospective de quatre patientes suivies au service de neurologie Sahloul à Sousse, ayant une atteinte distale motrice pure clinique et électromyographique sans topographie tronculaire ni signes centraux initiaux ou au cours de l’évolution. Les critères d'exclusion étaient une lésion médullaire à l'IRM, l'existence de blocs de conduction à l'EMG et une histoire familiale de maladie du motoneurone. Le diagnostic retenu était une MH après avoir éliminer les autres diagnostics différentiels d'un déficit moteur focal distal du membre supérieur sur les données de l'EMG et l'IRM médullaire [3].

## Résultats

**Figure 1:**
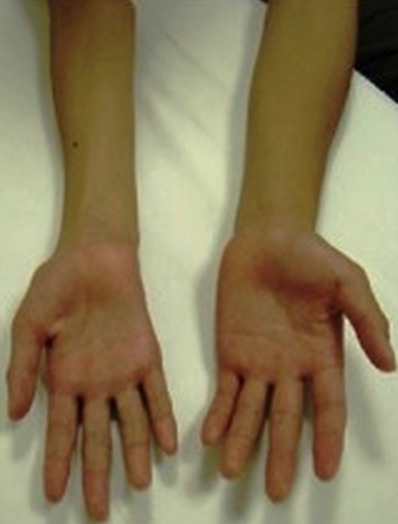
Amyotrophie des muscles de la main et de l'avant bras droits

**Figure 2:**
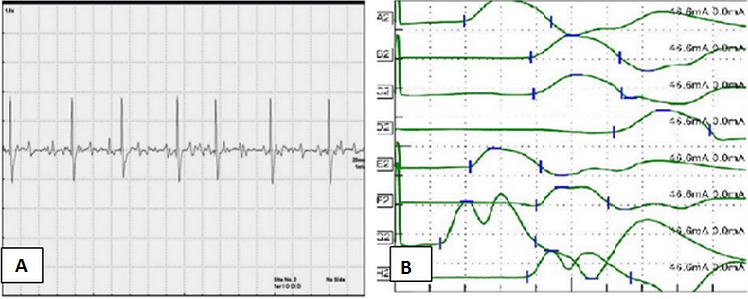
A: détection du premier interosseux dorsal de la main droite: tracé élémentaire; B: VCN motrices: la latence distale des nerfs médians et du cubital gauche est normale; celle du cubital droit est allongée (A2: médian proximal droit B2: médian distal droit C2: cubital proximal droit D2: cubital distal droit E2: médian proximal gauche F2: médian distal gauche G2: cubital proximal gauche H2: cubital distal gauche)

Il s'agit de quatre femmes S.C, I.B, H.M et A.K, issues d'un mariage non consanguin, âgées respectivement de 27, 28, 29 et 37 ans. La patiente S.C avait un antécédent de traumatisme cervical et trois avaient une profession (couturière) favorisant la position prolongée du rachis cervical en flexion. La patiente I.B appartenait à une équipe professionnelle de football. Avant la prise en charge de madame I.B dans notre service, elle a réalisé un EMG qui avait opté pour un syndrome du canal carpien bilatéral et un syndrome du canal de Guyon droit. Elle a été alors proposée pour la chirurgie et opérée des deux mains. Devant la non amélioration en post opératoire, elle avait consulté. La plainte initiale de toutes les patientes était un déficit moteur de la main droite d'installation progressive sur plusieurs années gênant l'activité quotidienne et professionnelle. L'examen neurologique à l'admission, retrouvait chez les quatre patientes un déficit moteur distal droit intéressant les muscles de l'avant bras (muscles extenseurs et fléchisseurs des doigts, muscles fléchisseurs du carpe et muscles extenseurs du carpe) et les muscles de la main (muscle opposant du pouce et les interosseux). Il s'y associait une amyotrophie des loges thénar, hypothénar ainsi que des muscles interosseux du coté droit (Figure 1). Les réflexes ostéotendineux y compris dans les territoires atteints étaient présents et symétriques. Le reste de l'examen neurologique était sans anomalie. Le bilan biologique (NFS, VS, glycémie, bilan lipidique, bilan hépatique, fonction rénale et LCR) et l'IRM médullaire étaient normaux. L'EMG avait objectivé des signes de dénervation motrice pure sévère avec des signes de perte axonale au niveau des territoires des racines C7, C8 et D1 (Figure 2). Toutes les patientes ont été mises sous vitaminothérapie associée à une rééducation motrice. L’évolution a été marquée par la stabilisation du tableau clinique avec un recul en moyenne de trois ans chez trois patientes. Une bilatéralisation de l'atteinte n’était observée que chez madame H.M.

## Discussion

L'atrophie musculaire spinale juvénile non progressive des membres supérieurs, l'amyotrophie brachiale monomélique et l'atrophie focale bénigne désignent la même pathologie [1–4]. Il s'agit d'une maladie rare qui touche préférentiellement l'homme jeune avec un pic de fréquence entre 15 et 17 ans et un sexe ratio H/F de 20:1 [4]. Le tableau clinique associe un déficit moteur de la main et de l'avant bras avec une amyotrophie manifeste sans atteinte des voies longues. Selon Wang et al, l'atteinte commence habituellement du coté droit [5]. Une atteinte bilatérale et asymétrique n’élimine pas le diagnostic et peut se voir dans le cadre d'une forme sévère de MH [3]. Les quatre patientes présentaient le même tableau clinique qui correspond à celui décrit dans la littérature. La patiente I.B a été opérée pour un syndrome de canal carpien. Le diagnostic de MH a été retenu en post opératoire devant la non amélioration clinique, les données électrophysiologiques et radiologiques. L'atteinte exclusive des femmes dans cette série pourrait être expliquée par les conditions de leur travail. Il a été suggéré que la MH pourrait être causée par une atteinte vasculaire de la corne antérieure de la moelle épinière s’étendant à la hauteur de C5 à T1 [3, 4]. Elle est favorisée par un traumatisme antérieur ou un exercice vigoureux du cou en flexion [5, 6]. Dans une étude sur 73 patients atteints de MH, 30 parmi eux avaient des antécédents de travail physique lourd ou d'entraînement physique [5]. D'ailleurs, une parmi nos patientes appartenait à une équipe professionnelle de football. Trois patientes travaillaient comme couturière pendant plusieurs heures durant plusieurs années. Les conditions de travail nécessitaient une flexion prolongée du cou.

Le diagnostic positif de MH se base sur les données cliniques et paracliniques. L'EMG est l'examen clé. Il montre des signes de dénervation motrice pure des territoires des racines C7, C8 et D1 [5]. La MH est caractérisée par l'absence d'anomalies significatives à l’étude de la conduction sensitive en particulier dans les régions amyotrophiées, la réduction de l'amplitude des réponses motrices proportionnelle à l'atrophie musculaire et une conduction motrice normale ou légèrement ralentie en rapport avec la perte des axones moteurs à conduction rapide [7]. Ces aspects électriques ont été constatés chez les quatre observations. Les tracés de détection musculaire montrent le caractère neurogène chronique qui touche essentiellement les muscles amyotrophiés [5, 7]. Il est possible que les anomalies électrophysiologiques intéressent des territoires apparemment intacts [7]. Dans toutes les observations aucune anomalie radiologique n'a était détectée. Dans la littérature, l'IRM médullaire en position neutre peut être normale ou montrer: une anomalie de courbure du rachis cervical, une atrophie médullaire en regard des espaces C5-C6 avec un aplatissement antéropostérieur de la moelle, un hypersignal médullaire antérieur et un défaut d'accolement postérieur du sac dural aux lames vertébrales [3, 8, 9]. L'IRM cervicale en flexion est recommandée en cas de suspicion de MH quand une IRM réalisée en position neutre est considérée comme normale [2, 4]. Elle peut mettre en évidence un déplacement antérieur du sac dural postérieur responsable d'une compression médullaire cervicale inférieure contre le mur vertébral postérieur et un élargissement de l'espace épidural postérieur au sein duquel apparaissent des veines dilatées [2, 8]. Nous ne disposons pas de plateau technique pour la réalisation de l'IRM en flexion. Le diagnostic de MH reste un diagnostic d’élimination. Le diagnostic différentiel se pose avec la sclérose latérale amyotrophique juvénile, la myélopathie cervicale traumatique vasculaire ou inflammatoire, la tumeur médullaire et la syringomyélie [3, 8]. L'atteinte myogène ou tronculaire doivent être écartées par l'EMG [3]. Tous ces diagnostics différentiels ont été éliminés chez les quatre patientes comme ont témoignent les données de l'EMG et l'IRM médullaire. Il s'agit d'une affection relativement bénigne puisque après une installation progressive sur deux à trois ans la maladie se stabilise réalisant une atrophie monomélique d'un membre supérieur [2]. Le traitement de la MH est généralement limité mais certains auteurs ont montré un effet bénéfique du port d'un collier cervical limitant la flexion du cou et/ou de la kinésithérapie à un stade initial de la maladie [6]. Certaines équipes ont montré l'efficacité d'une duroplastie, d'une décompression cervicale antérieure ou d'une reconstruction par transfert tendineux selon les cas sélectionnés [10]. Dans notre étude, toutes les patientes ont bénéficiées d'une kinésithérapie et d'une vitaminothérapie. L’évolution était marquée par la stabilisation clinique mais c'est l’évolution naturelle de la MH [2].

## Conclusion

La MH est une pathologie rare qui pose des problèmes de diagnostic positif et différentiel. Sa connaissance s'impose puisqu'il s'agit d'une maladie d’évolution bénigne qui prête à confusion avec les autres atteintes de la corne antérieure de pronostic sombre notamment la sclérose latérale amyotrophique.
